# A Review of Computational Tools in microRNA Discovery

**DOI:** 10.3389/fgene.2013.00081

**Published:** 2013-05-15

**Authors:** Clarissa P. C. Gomes, Ji-Hoon Cho, Leroy Hood, Octávio L. Franco, Rinaldo W. Pereira, Kai Wang

**Affiliations:** ^1^Institute for Systems BiologySeattle, WA, USA; ^2^Pós-Graduaçao em Ciências Genômicas e Biotecnologia, Universidade Católica de BrasíliaBrasília, Brazil; ^3^Centro de Análises Proteômicas e Bioquímicas, Pós-Graduação em Ciências Genômicas e Biotecnologia, Universidade Católica de BrasíliaBrasília, Brazil

**Keywords:** isomer, machine learning, miRNA conservation, RNA secondary structure, sequence homology

## Abstract

Since microRNAs (miRNAs) were discovered, their impact on regulating various biological activities has been a surprising and exciting field. Knowing the entire repertoire of these small molecules is the first step to gain a better understanding of their function. High throughput discovery tools such as next-generation sequencing significantly increased the number of known miRNAs in different organisms in recent years. However, the process of being able to accurately identify miRNAs is still a complex and difficult task, requiring the integration of experimental approaches with computational methods. A number of prediction algorithms based on characteristics of miRNA molecules have been developed to identify new miRNA species. Different approaches have certain strengths and weaknesses and in this review, we aim to summarize several commonly used tools in metazoan miRNA discovery.

## Introduction

The roles of microRNAs (miRNAs) in regulating biological processes through reshaping cellular transcriptome and proteome have been an exciting field since they were discovered in metazoan in 1993 (Lee et al., 1993). These small RNA molecules act through negatively regulating transcript levels post-transcriptionally, although positive regulation has been described in some cases (Vasudevan et al., 2007; Orom et al., 2008). MiRNAs also interact with protein translation machinery to attenuate the protein synthesis. It is estimated that miRNAs may regulate over 60% of transcripts in humans (Friedman et al., 2009), where each miRNA might target several messenger RNAs (mRNAs) and a single mRNA can also be targeted by several miRNAs (Brennecke et al., 2005; Lewis et al., 2005).

Technological advances such as bioinformatics and next-generation sequencing (NGS) allowed the identification of a great number of additional putative miRNAs in different organisms in recent years. Although it is difficult to estimate the total number of miRNAs in humans and other species (Berezikov et al., 2006), to date, over 2,000 human miRNAs have been identified and deposited in miRBase (release 19^1^) (Griffiths-Jones et al., 2008). However, the process of identifying miRNAs is still a complex and difficult task requiring interdisciplinary strategies, including the integration of experimental approaches with computational methods. A number of miRNA prediction algorithms have been developed, nonetheless the results merely provide a list of miRNA candidates, which requires extensive experimental works to identify real miRNAs in cells (Liu et al., 2012). In order to increase the accuracy of miRNA prediction, several characteristics of these regulatory RNA molecules have been taken into account.

Furthermore, since miRNAs differ between kingdoms, distinct approaches that take such characteristics into account have been applied (Mendes et al., 2009). For example, when compared to animal miRNAs, the stem-loop structures in plant miRNAs are more variable in size, being usually larger, they show more pairing between the miRNA and the other arm of the stem-loop, and tend to have around 21 nucleotides in length (Lau et al., 2001; Reinhart et al., 2002; Bartel, 2004). Despite applying similar principles to plant and animal miRNA prediction, fewer methods have been proposed to find new miRNAs in plants, in part, due to the heterogeneous nature of plant miRNA stem-loops (Mendes et al., 2009). On the other hand, several tools can be used to predict miRNAs both in animals and plants. In this review we focused on the strengths and weaknesses of tools used in animal miRNA discovery.

## MiRNA Biogenesis and Structure

Understanding the miRNA biogenesis is essential to build effective predictive models. Hence, some key points are important to briefly mention here (thoroughly reviewed in Krol et al., 2010; MacFarlane and Murphy, 2010; Starega-Roslan et al., 2011). Mature miRNAs are usually around 22 nucleotides long and a number of them are highly conserved throughout evolution (Bartel, 2004). They are located in either the introns of protein-coding genes or in the non-coding region of genes or intragenic regions of the genome (Lagos-Quintana et al., 2001; Lau et al., 2001; Lee and Ambros, 2001). MiRNAs derive from longer transcripts called primary miRNAs (pri-miRNAs), which are transcribed and processed similarly to protein-coding genes, with a 5′ cap and a 3′ poly-adenosine tail, but with hairpin structure(s). Such hairpin is characteristic of pri-miRNAs and is essential to be recognized and cleaved in the nucleus by the RNase III enzyme, Drosha. Its product is an approximately 70 nucleotides long hairpin precursor miRNA (pre-miRNA), which is then exported to the cytoplasm where it is further processed by Dicer, another RNase III enzyme, resulting in a double stranded ∼22 bp miRNA. Usually one of these strands produces the mature miRNA and will be associated to the RNA-induced silencing complex (RISC) to interact with its mRNA targets (Lee et al., 2002; Gregory et al., 2004).

In animals, the miRNA generally interacts with mRNAs through partial sequence complementation at the 3′-untranslated region (3′-UTR) of the transcripts, however, it has been documented that the 5′-UTR may also be targeted (Lee et al., 2009). It is due to this imperfect base pairing that one miRNA is able to have multiple mRNA targets and one transcript can be targeted by multiple miRNAs, thus forming a complex miRNA mediated gene regulatory network (Friedman et al., 2009). The down regulation of specific transcript levels is achieved through destabilization and degradation after miRNA-mRNA pairing (Filipowicz et al., 2008; Guo et al., 2010). Thus, generally a miRNA expression profile is negatively correlated with its targets expression profiles.

## Prediction of miRNA

The computational prediction of novel miRNAs revolves around miRNA gene identification. Different approaches have been adapted to identify putative miRNAs over the years. The traditional experimental method used to discover miRNAs consisted of cloning size-fractionated RNAs followed by Sanger sequencing and experimental validation. However, this procedure is time consuming, data is noisy (may clone and sequence a large number of degraded RNA fragments from the samples), and may not detect miRNAs that have low expression levels (Ambros et al., 2003; Mendes et al., 2009). Once these miRNAs were cloned, bioinformatic tools were required to locate their origin in the genome; sometimes this may not be a trivial task especially because eukaryotic genomes commonly present hairpin structures that are not necessarily related to miRNAs (Berezikov et al., 2006). Other experimental approaches have also been used to investigate new miRNAs such as Northern blot, microarray, and *in situ* hybridization, but these approaches are also tedious and time consuming (Table 1). The advent of NGS technology reduced the cost for discovery and offers significant advantage to identify lowly abundant miRNAs. It also provides a more reliable and sensitive method to quantify known miRNAs. However, to discover new miRNAs from NGS data, some kinds of miRNA prediction algorithms with proper computational infrastructure are required (Friedlander et al., 2008). Several computational tools have been developed to complement experimental approaches to identify and validate novel miRNAs from high throughput platforms such as NGS (Huang et al., 2011).

**Table 1:** **Experimental tools applied to discovery of miRNAs. NGS: next-generation sequencing**.

Technique used	Work reference	Organism
Cloning	Lee et al. (1993)	Nematode
	Lagos-Quintana et al. (2002)	Mouse
	Pfeffer et al. (2005)	Virus
	Bentwich et al. (2005)	Human
	He et al. (2007)	Rat
	Xu et al. (2009)	Bovine
	Long and Chen (2009)	
Microarray	Liu et al. (2004)	Human
		Mouse
*In situ* hybridization	Wienholds et al. (2005)	Zebrafish
	Kloosterman et al. (2006)	
	Nelson et al. (2006)	Human
	Deo et al. (2006)	Mouse
NGS	Bar et al. (2008)	Human
	Morin et al. (2008)	
	Friedlander et al. (2008)	Nematode
	Meng et al. (2012)	Rat
	Guzman et al. (2012)	Plant
	Kim et al. (2012)	

Among the main miRNA characteristics used by different computational tools are their length, high sequence conservation among species, and structural features like hairpin and minimal folding free energy (Li et al., 2010). Several algorithms to obtain putative secondary structure based on minimum free energy, like RNAfold and Mfold, have been used in miRNA identification (Hofacker, 2003; Zuker, 2003). Although cross-species homology is an effective and simpler criterion to discover new miRNAs, it misses the ability of finding non-conserved and species-specific miRNAs, such as the miR-466 cluster in mouse (Hertel and Stadler, 2006). Consequently, computational miRNA identification methods can be divided into two main strategies: comparative and non-comparative algorithms (Hertel and Stadler, 2006; Batuwita and Palade, 2009), although more specific and sub-classifications may be applied, once juxtapositions occur. A comparison between selected tools is summarized in Table 2.

**Table 2:** **Comparison of selected computational tools for miRNA prediction and their main characteristics**.

Tool	Website	Year	Conservation	Structure	Sequence	Machine learning	NGS^a^ application	Sensitivity (%)	Specificity (%)
miRscan	genes.mit.edu/mirscan	2003	X	X				74	40
miRSeeker	–	2003	X	X				75	
miRAlign	bioinfo.au.tsinghua.edu.cn/miralign	2005		X	X			90	98
Phylogenetic shadowing	–	2005	X					55	77
ProMiR	bi.snu.ac.kr/ProMiR	2005	X	X	X	X		73	96
Triplet-SVM	bioinfo.au.tsinghua.edu.cn/software/mirnasvm	2005		X	X	X		93	88
miR-abela	www.mirz.unibas.ch/cgi/pred_miRNA_genes.cgi	2005		X	X	X		71	91
RNAmicro	www.bioinf.uni-leipzig.de/~jana/index.php/jana-hertel-software/65-jana-hertel-rnamicro	2006		X	X	X		90	99
miRFinder	www.bioinformatics.org/mirfinder	2007	X	X		X		90	
miPred	www.bioinf.seu.edu.cn/miRNA	2007		X		X		84	98
MiRRim	www.ncrna.org/software/miRRim	2007	X	X		X		70	90
miRDeep	www.mdc-berlin.de/en/research/research_teams/systems_biology_of_gene_regulatory_elements/projects/miRDeep	2008		X			X	89	-
miRanalyzer	web.bioinformatics.cicbiogune.es/microRNA/miRanalyser.php	2009				X	X	98	
SSCprofiler	mirna.imbb.forth.gr/SSCprofiler.html	2009	X	X	X	X	X	89	84
HHMMiR	http://www.benoslab.pitt.edu/kadriAPBC2009.html	2009		X	X	X		84	88

*^a^NGS: Next-Generation sequencing*.

### Comparative methods

The nature of sequences conservation across different species for most of the known miRNAs led to the idea of using this unique characteristic to predict putative miRNA sequences from the non-coding regions of the genome. Sequence conservation of miRNAs may imply that they are involved in common biological processes, thus filtering for candidates with conserved sequences not only reduces the chances of finding false-positive miRNAs but may also indicate some common biological processes among different species (Lindow and Gorodkin, 2007). Although comparative methods considerably increased the number of predicted and validated miRNAs, it has low sensitivity for evolutionarily distant species and fail to detect species-specific miRNAs.

#### Closely related species conservation

Early computational methods for miRNA discovery generally focused on the secondary structure of RNA, looking for conserved hairpin structures between related species, such as srnaloop (Grad et al., 2003), MiRscan (Lim et al., 2003), and miRseeker (Lai et al., 2003). Srnaloop is a tool based on sequence conservation and structure similarity that was used to predict miRNAs from *Caenorhabditis* *elegans*. This algorithm is similar to BLAST (Altschul et al., 1990), however it supports alignments of shorter length and aligns complementary base pairs, like G-U, generating candidate miRNA hairpins (Grad et al., 2003).

However, MiRscan and miRseeker are more sensible tools, where both search for conserved intragenic region sequences that can form hairpin structures based on RNAfold (used in MiRscan) or Mfold (used in miRseeker). MiRscan then compares the identified structures with known miRNA features like 3′ and 5′-stem conservation, while miRseeker selects hairpins where the conservation patterns of nucleotide divergence were similar to those in the reference set (Terai et al., 2007). MiRscan was first applied to identify miRNAs in nematodes and miRseeker in flies, for which many predicted candidates were experimentally verified.

#### Multiple species conservation

A technique successfully employed by Berezikov et al. (2005) was called phylogenetic shadowing (Boffelli et al., 2003), which is based on short region sequence alignments to compare sequences of related species to determine the degree of sequence conservation of each nucleotide. One important finding of this work was the high degree of sequence conservation in the stems of miRNA hairpins, and the increased variation in nucleotides in the hairpin loops. Despite being able to find over 80% of human miRNAs that were known at the time with this algorithm, only 16 of the 69 predicted candidates could be experimentally validated by Northern blot. However, the poor validation rate may be simply due to the low expression levels for some of the predicted miRNAs in the samples (Berezikov et al., 2005).

#### Machine-learning approaches

Even though prediction algorithms based on machine-learning methods that don’t necessarily depend on sequence conservation were developed, many still chose to include it as one of the criteria. These methods use a set of known miRNAs (positive training dataset) and a set of sequences with hairpin structures that do not contain miRNAs, for example some mRNAs, tRNAs, and rRNAs (negative training dataset), to generate a predictor based on distinct properties that distinguish between presumed true and false miRNA sequences (Mendes et al., 2009).

These machine-learning based predictors generally take into account both sequence and structure features, such as minimum free energy of the hairpin structure, stem region sequence conservation, loop length, and inverted sequence repeat (Bentwich, 2005; Oulas et al., 2011; Williams et al., 2012). The results generated by machine-learning methods are then ranked by the degree of similarity based on the properties of known miRNAs (Bentwich, 2005; Mendes et al., 2009). Therefore, a careful choice of positive and negative datasets is crucial. Even though tRNAs and rRNAs have been used as negative training sets, we don’t known for sure whether hairpins from those RNAs cannot generate functional miRNAs (Allmer and Yousef, 2012). Therefore, one of the main drawbacks is the lack of good negative training sets (Yousef et al., 2008; Allmer and Yousef, 2012) and how the negative sequence dataset that was generated will affect the prediction results (Kim et al., 2006; Yousef et al., 2008). Methods that use only positive models to predict new miRNAs have been described (Wang et al., 2006; Yousef et al., 2008), since positive training data is more readily available. However, these methods are underperformed when compared to the two class approaches that utilize both positive and negative training sets (Yousef et al., 2008).

Machine-learning methods used to predict novel miRNAs include support vector machine (SVM), hidden Markov model (HMM), and naïve Bayes classifier. Several tools have been proposed based on these approaches to predict miRNAs from different species. RNAmicro (Hertel and Stadler, 2006) and MiRFinder (Huang et al., 2007), for example, are based on SVMs. HMM-based tools include ProMir (Nam et al., 2005), MiRRim (Terai et al., 2007), SSCprofiler (Oulas et al., 2009), and HHMMiR (Kadri et al., 2009). BayesMiRNAFind (Yousef et al., 2006) is an example of naïve Bayes classifier. While these techniques don’t require sequence conservation features, some chose to include them, such as RNAmicro, MiRFinder, ProMiR, and MiRRim. Like other methods using sequence conservation, it may hinder the identification of species-specific miRNAs (Huang et al., 2007).

RNAmicro combines comparative sequence analysis and structure prediction in order to find novel miRNAs. It uses predicted miRNA precursors from genome-wide survey results for non-coding RNAs generated by tools like RNAz (Washietl et al., 2005) and EvoFold (Pedersen et al., 2006). It then identifies “almost-hairpin” pre-miRNA candidates, builds descriptors of the candidates by considering structural and thermodynamic properties, and assesses the likelihood of descriptors as miRNA based on properties of known miRNAs to predict new ones. The design of RNAmicro aims for high sensitivity rather than minimizing the number of false positives (Hertel and Stadler, 2006).

MiRFinder compares sequences between related species and identifies hairpin structures from a set of miRNA candidates; it utilizes 18 different parameters, such as minimum free energy, base pairing of the hypothesized mature miRNAs, and frequency of possible secondary structure elements. Since there is a large number of sequences that can form pre-miRNA like hairpin structures, the method includes a randomization test to assess the statistical significance of the predicted structures, which significantly reduces the number of candidates, however, it might not detect species-specific pre-miRNAs (Huang et al., 2007).

ProMiR predicts miRNAs in close and distant homologs by sequence alignment using a probabilistic co-learning method based on HMM. It uses sequence and structure features of the pre-miRNAs, such as paired sequences in the stem region. Nine of the 23 predicted human miRNAs from ProMiR were validated by other experimental methods (Nam et al., 2005). MiRRim is also based on an HMM algorithm that considers both evolutionary and secondary structure properties of miRNA genes. It achieved high performance on the identification of new human miRNAs compared with other methods, especially for miRNA candidates that are clustered with known miRNAs (Terai et al., 2007).

### Non-comparative methods

Opposite to algorithms that adapt sequence conservation in the miRNA prediction, non-comparative methods do not rely on phylogenetic conservation and thus can find non-conserved or species-specific miRNAs (Batuwita and Palade, 2009). Non-comparative methods, which use intrinsic structural features of miRNA, include algorithms like PalGrade (Bentwich et al., 2005), Triplet-SVM (Xue et al., 2005), miPred (Ng and Mishra, 2007), miR-abela (Sewer et al., 2005), and HHMMiR (Kadri et al., 2009).

PalGrade was proposed by Bentwich et al. (2005) and combined computational predictions with microarray analysis and sequence-directed cloning. From an initial screen of around 11 million hairpin structure sequences identified in the human genome, this approach found 89 new miRNAs, from which 54 were primate-specific, among them 43 were validated by experimental methods (Bentwich et al., 2005).

Triptlet-SVM, presented by Xue et al. (2005), is based on a set of properties called the triplet elements to decipher contiguous local structure-sequence characteristics of miRNAs. For each nucleotide in a pre-miRNA sequence, there are only two possible structure statuses, paired or unpaired. Therefore, there are eight possible structure combinations for three adjacent nucleotides. Adding four different nucleotides, there will be 32 possible sequence structure combinations for any nucleotide with its immediate neighbors. These features for known miRNAs are extracted and an SVM is used to classify real and pseudo pre-miRNAs. For test data with known human miRNA from miRBase, an accuracy of 90% was achieved. Using the same human test data, the method was also applied to accurately classify pre-miRNAs in 11 other species, including plants, proving to be efficient to identify miRNA across different species, although with lower performance (Xue et al., 2005).

Another method that does not rely on phylogenetic sequence conservation is miPred, a tool based on random forest prediction model with characters from known pre-miRNAs at topological levels. The approach considers the dinucleotide frequencies, hairpin folding, and non-linear statistical thermodynamics; therefore, it relies on global and intrinsic properties of the RNA structure instead of specific regions of the sequence. The method achieved very high accuracy and sensitivity for human data (Ng and Mishra, 2007).

Sewer et al. (2005) developed a tool called miR-abela that is able to distinguish 40 pre-miRNA features, including folding free energy, length of stem and length of the hairpin loop, and then identifies miRNAs using an SVM. The method was successfully applied to find new mammalian (human, rat, and mouse) miRNAs by restricting to regions around known miRNA loci (clustered miRNAs). Although the specificity obtained was high, the sensitivity was low possibly because of an imbalance between the number of positive (178 human pre-miRNAs) and negative (5395 random transporter, ribosomal, and messenger RNA sequences) training sets. Nevertheless, several predicted miRNAs were confirmed (Sewer et al., 2005). Their results were comparable to tools relying on genomic conservation, such as phylogenetic shadowing (Berezikov et al., 2005) and a target-centered approach devised by Xie et al. (2005).

HHMMiR predicts miRNA hairpins without relying on evolutionary conservation by implementing a Hierarchical Hidden Markov Model (HHMM) that uses sequence and structure information of known pre-miRNAs. First it applies RNAFold to obtain secondary structures based on the minimum free energy. Then the algorithm extracts stem-loops and classifies with HHMM. HHMMiR was trained on human data, but it can be applied to other species like worm, flies, and plants. Despite depending on RNAFold results, an advantage of this method is that it examines each hairpin into four distinct regions, representing a better biological role of each region. Its average sensitivity was of 84% and specificity of 88% (Kadri et al., 2009).

#### Homology/secondary structure alignment

MiRAlign is a tool based on homology at both the structure and sequence levels and, like ProMiR, is an alignment method. One interesting feature of miRAlign is that it can use a looser sequence conservation requirement, revealing distant structure based homologs (Wang et al., 2005). This strategy provided better prediction results due to higher sensitivity than a similar method, ERPIN (Lambert et al., 2004). The potential drawback of miRAlign is that it is not able to detect new miRNAs with unknown structure homologs.

#### Tools directed to next-generation sequencing data

The commonly used tools among the methods directed to predict miRNAs from NGS data include miRDeep (Friedlander et al., 2008), miRanalyzer (Hackenberg et al., 2009), and SSCprofiler (Oulas et al., 2009). MiRDeep is able to discover previously known miRNAs as well as novel ones by using properties of miRNA biogenesis to score the compatibility of the position and frequency of sequenced RNA with the secondary structure of the pre-miRNAs (Friedlander et al., 2008). This method presumes that a true pre-miRNA has two distinct features, where higher number of reads correspond to the mature miRNA region of the stem-loop and less frequent reads correspond to other parts of the hairpin structure (Mendes et al., 2009). Originally, miRDeep used previously published data from *C. elegans* and generated RNA sequences from human and dog, predicting a total of 230 new miRNAs. Later works have applied miRDeep to predict novel miRNAs from other organisms like pig (Xie et al., 2011) and mouse (Inukai et al., 2012).

MiRanalyzer detects previously known miRNAs and employs a machine-learning algorithm to predict new miRNAs. The program detects known miRNAs annotated on miRBase; then it eliminates sequence matches, allowing no mismatches, to databases containing transcripts, such as mRNAs and other non-coding RNAs; and finally it predicts new miRNAs from the remaining sequences. Good performances were obtained, especially for *C. elegans*, rat, and human data (Hackenberg et al., 2009).

SSCprofiler uses sequence conservation to identify novel miRNAs and it may be applied to NGS data. Besides sequence conservation, it utilizes miRNA features such as secondary structure, employing a machine-learning algorithm to find miRNA precursors. This tool is similar to MiRRim in that it considers structure and conservation with an HMM algorithm (Oulas et al., 2011).

### Target-centered approach

A completely different approach to find novel miRNAs is using a reverse process: instead of starting with the RNA and/or DNA sequence, the procedure begins by finding potential conserved sequence blocks from mRNAs in order to infer potential miRNAs that might bind to them. This was first applied by Xie et al. (2005) and consisted of searching for frequent and highly conserved patterns in 3′-UTRs of protein-coding genes. Since such motifs on mRNAs may represent miRNA target sites, these were used to search for the corresponding pre-miRNA candidates.

Xie et al. found the existence of conserved sequence motifs in the 3′-UTR, which indicates the possibility of those sequences being involved in post-transcriptional regulations. Additionally, these motifs showed a different length distribution with a peak at an eight nucleotides in length, which tended to end with an adenosine. These are interesting findings because many mature miRNAs start with a uracil followed by a seven nucleotide seed that is complementary to the 3′-UTR of target mRNAs. By searching for complementary matches to the motifs of 8 bases (8-mer) in the human genome, Xie et al. were able to identify almost half of the known miRNAs at the time. To find new miRNAs based on these short motifs, they used sequence conservation across species combined with the ability to form hairpin structures (using RNAfold). From the small representative set of predicted miRNAs selected for confirmation, half was validated by different experimental methods (Xie et al., 2005).

Another work based on a similar reverse strategy was by Chang et al. (2008). The authors devised a computational pipeline to predict miRNAs from 3′-UTR motifs of human genes that are lowly or moderately expressed in selected tissues, called tissue-selective genes. They chose these genes from microarray and expressed sequence tag (EST) data and then identified frequent 7-mer sequence motifs in the 3′-UTRs of these genes as potential target sites of miRNAs. The predicted new miRNAs were inferred by the motifs that didn’t match any known miRNA seed region. They were able to validate the two predicted miRNAs that were tested (Chang et al., 2008).

Target-centered approaches rely on the identification of highly conserved motifs in the 3′-UTRs of protein-coding genes (Mendes et al., 2009), but as new mRNA target sites are described, such as 5′-UTRs (Lee et al., 2009), the search for conserved motifs should be expanded beyond the 3′-UTR regions (Li et al., 2010).

### Identification of individual miRNAs length and/or sequence heterogeneity

In the early days of miRNA profiling, the observed 5′ and/or 3′ length variation and even sequence changes were handled as errors or artifacts and were disregarded as an unexplainable phenomenon (Landgraf et al., 2007; Kim et al., 2008; Sdassi et al., 2009). However, the length and sequence variability were quickly recognized as part of the complexity behind the miRNA biogenesis and physiological role (Lee et al., 2010; Cloonan et al., 2011; Wyman et al., 2011). The length and sequence variants were named isomiRs (Morin et al., 2008). The functional evidences of isomiRs have been recently reviewed (Neilsen et al., 2012).

Since isomiRs were accepted as part of miRNA complexity, most of the NGS based profiling starts to report them (Chang et al., 2012; Ple et al., 2012; Polikepahad and Corry, 2013). Most of the articles deal with isomiRs using their own scripts (Zhou et al., 2012). Aiming to offer a source to catalog isomiRs, a database was compiled and made available^2^ (Lee et al., 2010). Recently this type of miRNA sequence variation has also been included in the miRBase. Cheng et al. (2013) developed the YM500, an integrated database predicted on results from 609 human and mice small RNA NGS data (Cheng et al., 2013). It allows the user to change options such as mismatches, 5′ and 3′ addition or trimming, and retrieve a list of isomiRs based on a canonical miRNA according to the selected options. The retrieved list can be used to compare against a specific dataset.

The computational tools discussed in the previous sessions of this review do not deal directly with isomiRs. In this regard, SeqBuster, a tool developed to process small RNA datasets, also considers isomiRs (Marti et al., 2010; Pantano et al., 2010). It can be used as a web tool or as a stand-alone user installed version. SeqBuster pipeline is based on two major processes: pre-analysis and analysis steps. During the pre-analysis, adapters are removed and reads are counted, mapped, and annotated. Several R-based packages allow basic analysis of processed and annotated small RNA dataset, which can also be performed with other tools described earlier in this review. The innovative feature in SeqBuster is the isomiR analysis. The R-packages implemented allow a deep analysis of isomiRs: distribution and statistical differences among different types of variability; percentage of a specific miRNA with a specific type of change; list of miRNAs that do not present any sequence variability. Comparative expression analysis can then be carried out at the isomiR level.

## Conclusion and Perspectives

With the advent of novel and powerful informatics infrastructure as well as biocomputational tools, the possibility to discover novel biomolecules and interactions in complex datasets became feasible. Recent studies have shed some light over the function of miRNA, which may lead to numerous potential applications from infectious diseases control, cancer development decrease, and inhibition of protein syntheses to improvement of plant production in the agribusiness (as revised by Pillai, 2005). Gaining some knowledge on the complete spectrum of miRNA is critical in understanding its function and developing miRNA based applications.

Even though methods, as summarized in this review, have been developed in an attempt to identify miRNAs, new algorithms are still in need to improve the ability to find new miRNAs and relate them with their respective functions. For instance, an accurate practical comparison between the methods is difficult because they use different datasets to generate the results and are not always based on the same parameters to evaluate their performance. Therefore, the selection of a methodology to use for a study probably depends on the informaiton available. Many tools still generate a great number of false positives and don’t provide insights into the function or regulatory role of the predicted candidates. The lack of a clear and simpler pipeline to predict and validate miRNA candidates also makes the task of predicting miRNA transcripts and its encoded miRNAs complicated.

Taking in consideration the steps along the process of miRNA maturation could improve such miRNA prediction; however we do not completely understand various steps involved, like how the strand selection of the mature miRNA is decided. Thus, the improvement of computational tools is tightly linked to experimental biological research and vice versa. For example, the comprehension that isomiRs are not artifacts from experimental approaches should be incorporated into prediction tools. With improvement in precision and accuracy of miRNA prediction, the number of miRNAs might possibly be increased significantly, which may provide more comprehensive understanding on miRNA mediated gene/protein regulation.

## Conflict of Interest Statement

The authors declare that the research was conducted in the absence of any commercial or financial relationships that could be construed as a potential conflict of interest.

## Footnotes

^1^www.mirbase.org

^2^http://galas.systemsbiology.net/cgi-bin/isomir/find.pl
